# The real bacterial filtration efficiency to evaluate the effective protection of facemasks used for the prevention of respiratory diseases

**DOI:** 10.1038/s41598-023-35071-1

**Published:** 2023-06-05

**Authors:** Pedro J. Benito, Álvaro Gutiérrez, Miguel A. Rojo-Tirado

**Affiliations:** 1 LFE Research Group, Department of Health and Human Performance, Faculty of Physical Activity and Sports Science, Universidad Politécnica de Madrid, 28040 Madrid, Spain; 2 ETSI Telecomunicación, Universidad Politécnica de Madrid, Av. Complutense 30, 28040 Madrid, Spain

**Keywords:** Environmental sciences, Health care

## Abstract

The real protection offered by facemasks to control the transmission of respiratory viruses is still undetermined. Most of the manufacturing regulations, as well as scientific studies, have focused on studying the filtration capacity of the fabrics from which they are made, ignoring the air that escapes through the facial misalignments, and which depends on the respiratory frequencies and volumes. The objective of this work was to define a Real Bacterial Filtration Efficiency for each type of facemask, considering the bacterial filtration efficiency of the manufacturers and the air that passes through them. Nine different facemasks were tested on a mannequin with three gas analyzers (measuring inlet, outlet, and leak volumes) inside a polymethylmethacrylate box. In addition, the differential pressure was measured to determine the resistance offered by the facemasks during the inhalation and exhalation processes. Air was introduced with a manual syringe for 180 s simulating inhalations and exhalations at rest, light, moderate and vigorous activities (10, 60, 80 and 120 L/min, respectively). Statistical analysis showed that practically half of the air entering to the system is not filtered by the facemasks in all intensities (p < 0.001, ηp^2^ = 0.971). They also showed that the hygienic facemasks filter more than 70% of the air, and their filtration does not depend on the simulated intensity, while the rest of the facemasks show an evidently different response, influenced by the amount of air mobilized. Therefore, the Real Bacterial Filtration Efficiency can be calculated as a modulation of the Bacterial Filtration Efficiencies that depends on the type of facemask. The real filtration capacity of the facemasks has been overestimated during last years since the filtration of the fabrics is not the real filtration when the facemask is worn.

## Introduction

The use of facemasks is one of the most widely employed non-pharmacological interventions by all health policies worldwide, along with social distancing and hand hygiene, to reduce the transmission of all types of viruses^1^. This transmission mainly occur through the mouth, nose or eyes via respiratory droplets, aerosols or fomites^2,3^, such as severe acute respiratory syndrome coronavirus 2 (SARS-CoV-2), causing coronavirus disease 2019 (COVID-19), which has infected more than 512 million people^4,5^.

Therefore, face masks have been used by global health agencies and countries in the world to minimize the risk of respiratory droplets reaching the nasal or oral mucosa of others^6^, although their recommendations vary^7^. In fact, the World Health Organization acknowledges that there is no evidence that wearing a facemask protects healthy people from SARS-CoV-2, as it has been recently demonstrated in a randomized clinical trial^1,8^, Specifically, comparisons between N95 and medical masks did not show any statistically difference on viral infection transmission^9^. Moreover, wearing a medical mask by healthy individuals has not shown evidence of reducing transmission of illness in households with a SARS-CoV-2 inhabitants^10^. Furthermore, specific studies compared health care workers wearing and not wearing a mask, showing no statistically significant reduction of respiratory viruses’ propagation^11,12^.

It is understood that reducing the release of virus from infected persons into the environment may be the mechanism to mitigate transmission in communities where facemask use is common or mandatory, provided that the physical properties of their materials ensure proper air filtration, according to UNE 0065:2021, UNE-EN 14683:2019 + AC:2019, UNE-CWA 17553:2020 or UNE-EN 1827:1999 + A1:2010; and its facial adjustment is appropriate for each individual to reduce the probability of unfiltered air leakage. Most studies that looked at filtration efficiency examined the ability of different layers of respirators to filter particulates, bacteria, viruses, and NaCL^2,3,13^. Some other relied on the negative or positive pressure to study how well the mask or respirator fits an individual facepiece^3,5^. Several studies quantified mask fit by simultaneously measuring particle concentrations inside and outside the mask on a second-by-second basis with linear regression models^4,7^ without determining the amount of particulate matter that is filtered by the tissue or that is leaked by different facial mismatches of the mask. Precedents already exist for the study of air leakage in facemasks^1,4^. However, this work was not aimed at analyzing these leaks, but rather the performance of four fans of a pneumotachograph coupled to a differential pressure transducer^14^. Currently, the Flow Tester for UNE-EN 14683 High Level device, marketed by Fortest (https://www.fortest.es/es/productos/c/gama-t/p/t9731), equipped with air flow meters and a double differential pressure manometer, performs evaluations according to the UNE-EN 14683 Standard, without quantifying air leakage. A recent study determined a new technique for obtaining the filtration properties of facemask fabric, using ultrasonic waves^15^. In terms of leakage, although there is a 2010 study that showed interest in measuring leakage^14^, there is no study in the literature that proposes a methodology to measure possible leaks through a validated procedure and compares the types of facemasks most commonly used today. Thus, we still do not know the real protection coefficient offered by each facemask, since the standards are limited to assessing the filtration capacity of each material, ignoring the air that leaks and is not filtered, raising the hypothesis that perhaps we are not measuring well the protection capacity of facemasks worldwide and that the design and manufacturing standards should be reconsidered. Therefore, the objective of this work was to create a Real Bacterial Filtration Efficiency for each type of facemask, considering the bacterial filtration efficacy of the manufacturers and the air that passes through each type of facemask, for a wide range of facemasks available to the world’s population.

## Materials and methods

### Study design

The present study simulates the breathing pattern model that occurs in different circumstances of the daily life of humans, under resting conditions and with progressive increases in the intensity of physical activity. We have used a crossover design, where each facemask was tested on five consecutive occasions, leaving a rest of five minutes between tests, and recording all environmental data, such as temperature, air humidity, atmospheric pressure, and environmental CO_2_.

**Figure 1:**
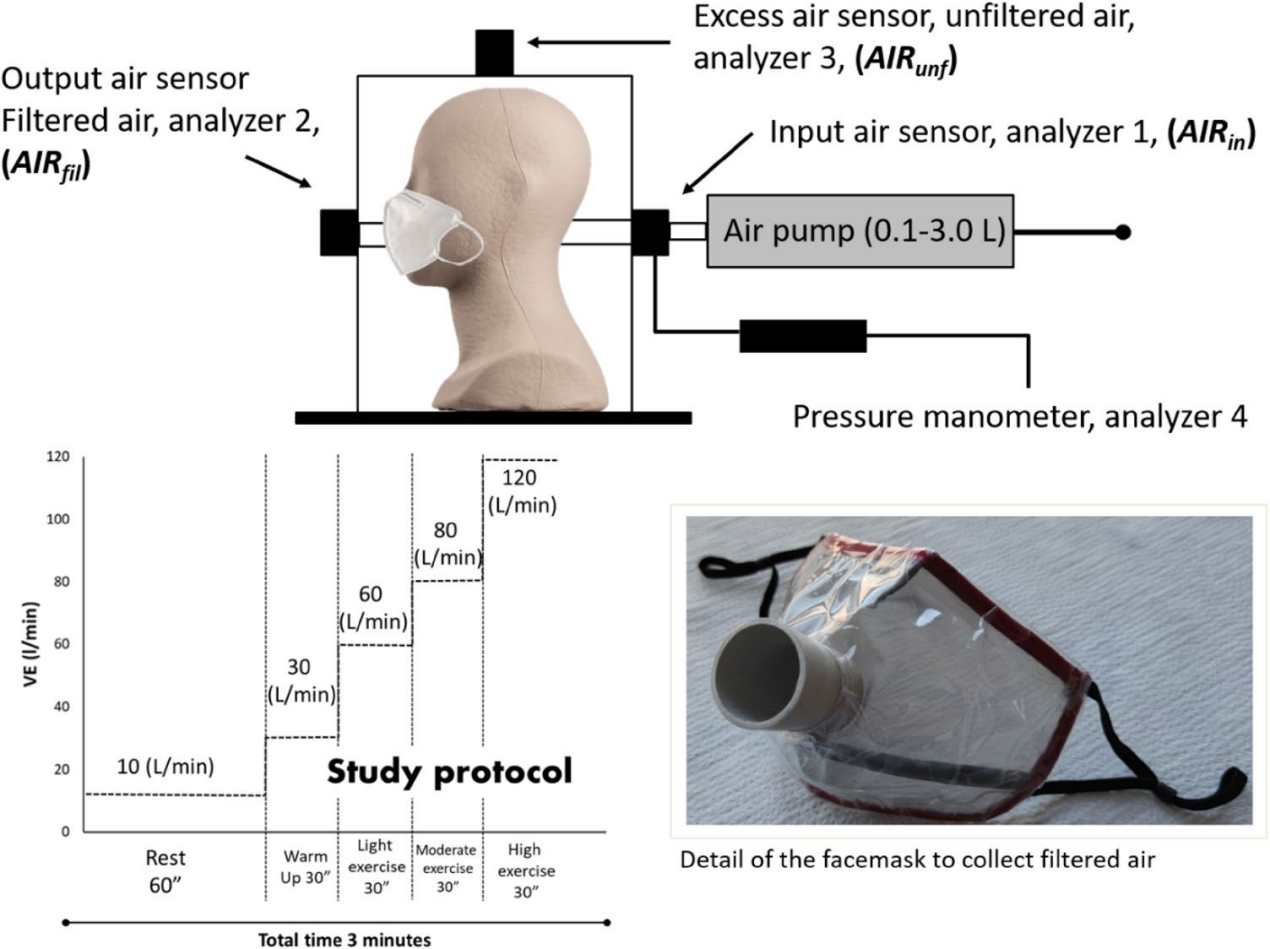
Design of the protocol and measurement equipment, and detail of the filtered air collection procedure.

A 3 mm thick polymethylmethacrylate box measuring 320 × 300 × 300 mm (height × length × width) was designed and used for the study. Three 30 mm radious apertures were made in it to attach a volume sensor, from a gas analyzer, in each one of them. Three gas analyzers were used to measure the breathing pattern variables analyzed: the Jäeger Oxycon Mobile^®^ (Jäger, Würzburg, Germany), which was placed in the “air input” just behind the calibration pump (Analyzer 1 see Fig. 1), and measured the “air input” to the system (*AIR*_*in*_); the Jäeger Oxycon Pro^®^ (Jäger, Würzburg, Germany), which was placed at the front “air output” of the facemassk (Analyzer 2), and measured the air filtered by the mask (*AIR*_*fil*_); and finally, the Vyntus CPX (Vyaire, Mettawa, Illinois, USA), at the top of the box, which collected the air that was not filtered (*AIR*_*unf*_) (Analyzer 3). The reliability found in the ventilation measurement of the three analyzers placed in line behind the calibration pump showed an intraclass correlation coefficient of R = 0.999 with p < 0.001, a standard error of 1.09 L/min and a percentage error of 2.1% (see Supplementary data).

The air pressure measurement was performed with a digital pressure manometer MAN-37 (Kowloon, Hong Kong) with a differential pressure gauge (Analyzer 4 see Fig. 1), that enabled the pressure data to be exported to a text file with a sampling frequency of 1 Hz.

### Facemasks

We tested nine different facemasks from seven different manufacturers. Facemasks description and available information is listed in Table 1. In general, facemasks were made of two external and very thin layers and between then one to three inner layers. One difference observed is that reusable facemasks are stretchable. Therefore, it can be anticipated that any stretching of the ear loops (either during use or during testing) may affect the properties of the facemask. To avoid this, during the tests, an attempt was made to maximize the degree of adequacy of the facemasks with the surface of the face.

**Table 1:** Description of the facemasks used in this study.

Name	Description	BFE (%)	Manufacturer/other information	Number of trials
Surgical_1 (MVT)	Three layers. UNE 0064-1:2020	> 95	Foshan Boentai Commodity Co., Ltd	5
Surgical_2 (Radex)	Three layers. EN 14683:2019 + AC:2019	> 98	Grafoplas del Noroese, S.A	5
Hygienic_1, reusable (Emotion)	One layer. EN 14683:2019 + AC:2019, ISO 9237, ISO 14184-1; ISO 3071	95	Texcon y Calidad^®^, Spain, Size “M”	Test 5–retest 5
Hygienic_2, reusable (Elite)	One layer. EN 14683:2019 + AC:2019, ISO 9237, ISO 14184-1; ISO 3071	95	Texcon y Calidad^®^, Spain, Size “M”	5
Hygienic_2, reusable (LifeStyle)	One layer. EN 14683:2019 + AC:2019, ISO 9237, ISO 14184-1; ISO 3071	95	Texcon y Calidad^®^, Spain, Size “M”	5
FFP2 Aura	FFP2-NR, four layers, EN149:2001, CE2797	94	Aura TM 9320+, by 3M	5
FFP2 Palens	FFP2, three layers PP/PP/nanofiber layer (external), EN 149: 2001 + A1: 2009, COVID-19 CE 0370-4080-PPE/B	96.4	Palens PLNS1620, Palens Barcelona, S.L	5
FFP2 Biofield	FFP2-NR, five layers EN149:2001 + A1:2009 CE2163	> 94	Biofield, by Zhejiang Lily	5
FFP3 MC002	FFP3-NR, five layers EN149:2001 + A1:2009 CE2163	> 98	Quanzhou City Meichen Protective Products Co., Ltd	5

*BFE* bacterial filtration efficiency.

Each mask was covered with a 0.1 mm thick plastic wrap and high adhesion 3 M double-sided tape, leading to a 30 mm diameter cannula through which all the air filtered by the mask was collected (see Fig. 1). The 30 mm cannula was positioned at the exit hole. In the supplementary material, the results of performing five measurements without changing this wrap versus changing the wrap each time can be observed.

### Protocol of test

Before each trial, the adjustment of the facemask and all the measuring elements were checked, and the environmental conditions were noted. The protocol was started by simulating ventilation at rest for 60 s (~ 10 L/min)^16^. Without pauses between any phase, the next phase started pumping about 30 L/min, named the “warm-up phase”, with a duration of 30 s. In the next three phases (“light, moderate and high intensity exercise”), 60, 80 and 120 L/min of air were pumped, respectively, with a duration of 30 s each. Therefore, the total duration of the protocol was 180 s (see Fig. 1). In total, 45 experiments were performed.

The data from each analyzer [ventilation (L/min), tidal volume (inspired/expired) (L), time (inspiratory/expiratory) (s) and breathing frequency (Hz)] were exported breath by breath and stored in separate files. Pressure data from each embolus of the syringe were exported every second (1 Hz) from the manometer. Of these, absolute values above the median were considered as expiratory values, while negative inspiratory values below the median were considered as expiratory values. Then, both gas analyzer and manometer data were combined into a single database for further analysis.

### Real bacterial filtration efficiency

Bacterial filtration efficiencies (BFE) were obtained from the technical specifications of each facemask manufacturer. However, to calculate the Real Bacterial Filtration Efficiency (RBFE) of each facemask, the BFE must be corrected by the real filtered air of each facemask (see Eq. 1).

1 $$ RBFE = \alpha \cdot BFE $$

where α is the correction coefficient obtained as a linear regression (see Table 4 of "Results").

The amount of air filtered in percentage (Fig. 3) by each facemask was calculated as the fraction of “input air” divided by the “output air” multiplied by 100.

### Statistical analysis

All values are expressed as mean ± standard deviation for tables and mean ± standard error of mean (SEM) for figures. A three-way ANOVA for repeated measures (5 × 3 × 9) was performed to analyze the effect of the five exercise phases (rest, warm-up, light exercise, moderate exercise and high exercise), three analyzers (*AIR*_*in*_, *AIR*_*fil*_ , *AIR*_*unf*_ ), and nine types of facemask (Surgical_1 (MVT), Surgical_2 (Radex), Hygienic_1 (Emotion), Hygienic_2 (Elite), Hygienic_3 (LifeStyle), FFP2_Aura, FFP2_Palens, FFP2_Biofield, FFP3_MC002) in the ventilation, tidal volume (inspired/expired), time (inspiratory/expiratory) and breathing frequency.

A three-way ANOVA for repeated measures (5 × 2 × 2) was performed to analyze the effect of the five exercise phases (rest, warm-up, light exercise, moderate exercise and high exercise), two breathing phases (inspired/expired air) and two analyzers (*AIR*_*in*_, *AIR*_*fil*_ ) for tidal volume variable. Similarly, a three-way ANOVA for repeated measures (5 × 2 × 4) was performed to analyze the effect of the five exercise phases (rest, warm-up, light exercise, moderate exercise, and high exercise), two breathing phases (inspired/expired air and four type masks (FFP2, FFP3, Hygienic and surgical) in the pressure variables.

To analyze the effect of changing the mask covering plastic on each occasion, the ventilations of the five attempts of the Emotion facemask without changing the plastic were compared to the five attempts where the plastic was changed on each occasion, using a T-Student for independent samples.

Mauchly’s sphericity test was carried out to evaluate whether the sphericity assumption of the variances was violated, in which case the Huynh–Feldt correction was applied. Bonferroni post-hoc tests were conducted, where significant differences were found in any of the analyzed factors.

Intraclass correlation coefficient was used for estimating the reliability with the ventilation measurement of the three analyzers placed in line behind the calibration pump.

A stepwise linear regression analysis was performed, with the dependent variable being filtered air and the independent variables being all the variables of the proposed respiratory model in all intensities (input air volume, output air volume, respiratory times, pressures, etc.).

Effect size (ES) was estimated by partial eta-squared (ηp^2^) and considering effects > 0.2 small, > 0.5 medium and > 0.8 large. Data were analyzed using the SPSS Statistic software, version 26.0 for Windows (IBM Corporation; Armonk, New York). The significance level was set at p < 0.05.

## Results

When all the facemasks were analyzed together, it can be observed that for all the variables studied in the breathing pattern model there was a double interaction between the phase of the protocol and the analyzer, although not equally for all variables (see Table 2). In this sense, ventilation showed an interaction between these two factors in all intensities, (F_(5,3)_ = 1.208, p < 0.001, ηp^2^ = 0.971), showing that almost half of the air entering the system is not filtered by the facemasks.

**Table 2:** Ventilatory results foreach exercise intensity phase measured from the three gas analyzers. n = 45.

	Exercise intensity phase	Rest (10 L/min)		Warm-up (30 L/min)		Light intensity exercise (60 L/min)		Moderate intensity exercise (80 L/min)		High intensity exercise (120 L/min)	
		Mean	SD	Mean	SD	Mean	SD	Mean	SD	Mean	SD
VE (L/min)	Input air	12.3	1.4	36.3	4.7	61.3	7.2	84.2	5.0	120.9	4.9
	Output air (filtered)	5.4^a^	3.2	17.2^a^	9.0	28.1^a^	14.6	37.8^a^	21.0	50.6^a^	32.3
	Excess air (unfiltered)	6.8^ab^	3.6	17.6^a^	8.5	30.7^a^*	15.4	44.7^a^*	21.4	65.4^ab^	30.8
VT in (L)	Input air	0.432	0.067	0.965	0.120	1.379	0.183	1.706	0.193	2.093	0.186
	Output air (filtered)	0.279^a^	0.081	0.593^a^	0.173	0.953^a^	0.166	1.252^a^	0.185	1.541^a^	0.238
	Excess air (unfiltered)	0.267^a^	0.131	0.400^ab^	0.199	0.472^ab^	0.233	0.534^ab^	0.246	0.619^ab^	0.260
VT ex (L)	Input air	0.471	0.077	1.040	0.136	1.510	0.211	1.900	0.225	2.357	0.187
	Output air (filtered)	0.212^a^	0.085	0.451^a^	0.215	0.628^a^	0.307	0.758^a^	0.404	0.886^a^	0.576
	Excess air (unfiltered)	0.268^ab^	0.109	0.484^a^	0.235	0.709^ab^	0.366	0.920^ab^	0.466	1.146^ab^	0.548
T in (s)	Input air	1.06	0.14	0.84	0.11	0.75	0.12	0.68	0.11	0.61	0.09
	Output air (filtered)	1.14	0.32	0.88	0.18	0.76	0.13	0.69	0.12	0.62	0.09
	Excess air (unfiltered)	1.66^a^	1.08	0.92^a^	0.14	0.74	0.13	0.67	0.12	0.60	0.09
T ex (s)	Input air	1.07	0.14	0.79	0.12	0.63	0.11	0.54	0.08	0.44	0.09
	Output air (filtered)	1.79^a^	1.09	0.82^a^	0.15	0.64	0.12	0.53	0.09	0.44	0.10
	Excess air (unfiltered)	1.14**	0.29	0.82^a^	0.12	0.67^ab^	0.11	0.56	0.08	0.46^ab^	0.09
Tt (s)	Input air	2.13	0.26	1.63	0.21	1.38	0.18	1.22	0.12	1.04	0.09
	Output air (filtered)	3.00^a^	1.28	1.71^a^	0.29	1.40^a^	0.18	1.22	0.12	1.05	0.08
	Excess air (unfiltered)	2.79^a^	1.07	1.74^a^	0.22	1.41^a^	0.18	1.23	0.12	1.06	0.09
BF (Hz)	Input air	28.9	3.2	37.8	4.7	44.6	5.2	49.8	4.7	58.2	4.9
	Output air (filtered)	23.8^a^	7.2	36.9^a^	5.1	44.1	5.2	49.7	4.8	57.8	4.7
	Excess air (unfiltered)	24.5^a^	6.8	36.5^a^	4.2	43.7^a^	5.1	49.6	4.7	57.1^a^	4.8

*VE* ventilation, *VT* in tidal volume inspired, *VT ex* tidal volume expired, *T in* time inspired, *T ex* time expired, *Tt* total time, *BF* breathing frequency.

*Significant differences with input air (p < 0.05).

**Significant differences with output air (p < 0.05).

^a^Significant differences with input air (p < 0.001).

^b^Significant differences with output air (p < 0.001).

When analyzing the effect of changing the plastic wrap that covers the facemask for each measurement versus making the 5 measurements with the same plastic for the Hygienic_1, reusable (Emotion) facemask, no significant differences were found in any of the three analyzers (*AIR*_*in*_*, AIR*_*fil*_ , *AIR*_*unf*_ ) (see Supplementary data).

**Figure 2:**
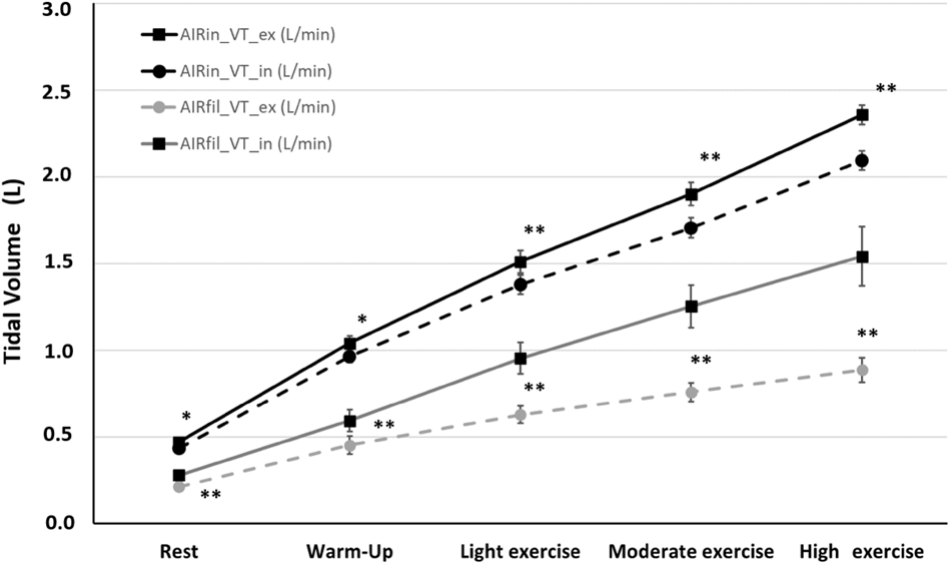
Tidal volume, comparing the five intensities, mask input and output air (AIR_*in*_ vs AIR_*fil*_) and inspiratory and expiratory tidal volumes of all masks together. *Significant differences between inspiratory and expiratory values (p < 0.05), **significant differences between inspiratory and expiratory values (p < 0.001).

Tidal volume, both inspiratory (F_(5,3)_ = 840, p < 0.001, ηp^2^ = 0.959) and expiratory (F_(5,3)_ = 784, p < 0.001, ηp^2^ = 0.956) showed a similar response to human ventilation. A three-way interaction was found in the tidal volume specific analysis of variance, comparing the five intensities, “air input” and “output air” and inspiratory and expiratory tidal volumes (F(_5,2,2_) = 85.9, p < 0.001, ηp^2^ = 0.494) (see Fig. 2).

However, the variables that are related to respiratory times showed a different response than volume variables. Both inspiratory time (F_(5,3)_ = 68, p < 0.001, ηp^2^ = 0.654) and expiratory time (F_(5,3)_ = 60, p < 0.001, ηp^2^ = 0.625) showed greater differences in the phases where intensity is lower, tending to disappear when the intensities of the phases increase.

Total ventilation time (F_(5,3)_ = 65, p < 0.001, ηp^2^ = 0.643) and breathing frequency (F_(5,3)_ = 43, p < 0.001, ηp^2^ = 0.544) showed a very similar response, since one is the inverse of the other. However, this response tends to disappear as the phase intensity increases.

**Figure 3:**
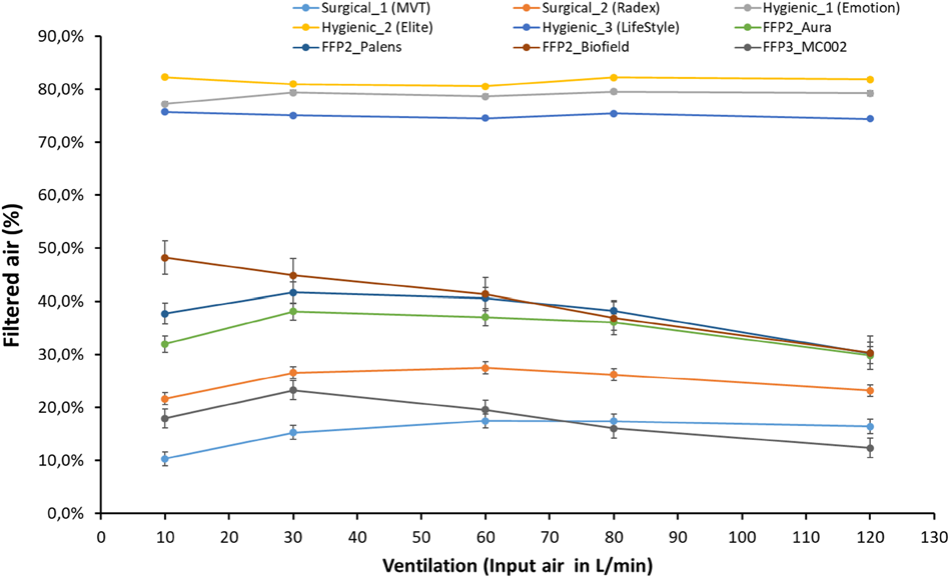
Ratio of air filtered by each facemask (air output/air input × 100), with respect to the air introduced by the pump at different intensities of the protocol. The points indicate the mean value for each facemask and the bars the statistical error of mean.

Specifically, analyzing ventilation and, therefore, the air that is filtered or not, in relation to the type of facemask, it can be observed that not all facemasks respond in the same way. A triple interaction was found between the factors intensity phase, analyzer and type of facemask for ventilation (F_(5,3,8)_ = 108, p < 0.001, ηp^2^ = 0.960) which is reflected in Fig. 3. It shows that the hygienic facemasks filtered more than 70% of the air, and their filtration did not depend on the intensity, while the rest of the facemasks showed an obviously different response. Table 3 indicates that, if we compare three groups of facemasks (surgical, hygienic and FFP), the hygienic facemasks were the ones that filtered the most air (78.5 ± 0.7%), not being influenced by the intensity of the exercise (Fig. 3), while the other facemasks were influenced by the amount of air mobilized. After the hygienic facemasks, the facemask that filtered the most air was the FFP2 Biofield. At rest, the FFP2 Biofield filtered around 48.5 ± 0.8%, while at high intensity it filtered no more than 30.4 ± 0.8% (see Fig. 3).

**Table 3:** Differences in input, output and excess air between facemasks. n = 5.

	Analyzer	Input air		Output air (filtered)		Excess air (unfiltered)	
	Facemask	Mean	SD	Mean	SD	Mean	SD
Rest	Surgical_1 (MVT)	13.5	0.8	1.4^bc^	0.5	11.9^bc^	1.0
	Surgical_2 (Radex)	12.8	1.7	2.8^bc^	0.7	10.0^bc^	1.8
	Hygienic_1 (Emotion)	11.7	1.3	9.0^ac^	0.8	2.4^ac^	0.3
	Hygienic_2 (Elite)	12.3	0.9	10.1^ac^	0.8	2.2^ac^	0.2
	Hygienic_3 (LifeStyle)	11.9	0.8	9.0^ac^	0.7	3.3^ac^	0.2
	FFP2_Aura	11.8	0.7	3.8^ab^	0.6	7.9*^b^	1.0
	FFP2_Palens	12.2	1.0	4.6^ab^	1.4	7.5*^b^	1.3
	FFP2_Biofield	11.0	1.0	5.3^ab^	0.6	5.1^ab^	0.6
	FFP3_MC002	13.5	2.1	2.4^bc^	1.0	10.6^bc^	1.7
Warm-up	Surgical_1 (MVT)	37.1	7.3	5.7^bc^	2.2	28.7^bc^	5.4
	Surgical_2 (Radex)	32.9	5.8	8.8^bc^	1.8	22.7^bc^	4.8
	Hygienic_1 (Emotion)	34.8	3.7	27.7^ac^	2.9	6.9^ac^	0.6
	Hygienic_2 (Elite)	37.2	3.4	30.1^ac^	3.0	5.9^ac^	0.5
	Hygienic_3 (LifeStyle)	35.5	1.8	26.7^ac^	1.4	8.8^ac^	0.5
	FFP2_Aura	37.4	4.3	14.3^ab^	2.6	21.8^b^	2.0
	FFP2_Palens	37.6	3.9	15.7^ab^	2.0	20.3*^b^	4.8
	FFP2_Biofield	39.0	7.0	17.5^ab^	3.1	18.9*^b^	3.2
	FFP3_MC002	35.4	3.7	8.2^bc^	3.5	24.9^b^***	3.6
Light exercise	Surgical_1 (MVT)	68.3	9.7	11.9^bc^	3.7	51.8^bc^	7.6
	Surgical_2 (Radex)	61.2	5.3	16.8^bc^	2.6	41.2^bc^	6.3
	Hygienic_1 (Emotion)	54.3	12.9	42.8^ac^	10.0	10.4^ac^	2.4
	Hygienic_2 (Elite)	61.2	1.5	49.3^ac^	1.8	9.6^ac^	0.5
	Hygienic_3 (LifeStyle)	63.9	2.7	47.6^ac^	1.9	15.3^ac^	0.9
	FFP2_Aura	59.1	7.9	21.9^ab^	3.0	34.3^ab^	5.2
	FFP2_Palens	59.7	4.6	24.2^ab^	3.4	32.6^ab^	5.7
	FFP2_Biofield	62.2	6.3	25.8^ab^	2.8	35.3^ab^	6.6
	FFP3_MC002	61.5	3.6	12.1^bc^	2.7	46.0^bc^	3.6
Moderate exercise	Surgical_1 (MVT)	91.7	4.2	16.0^bc^	5.8	70.6^bc^	5.0
	Surgical_2 (Radex)	86.4	2.5	22.7^bc^	3.3	61.4^bc^	4.8
	Hygienic_1 (Emotion)	78.9	9.5	62.8^ac^	8.0	15.6^ac^	2.2
	Hygienic_2 (Elite)	83.7	1.6	68.9^ac^	1.2	14.0^ac^	0.5
	Hygienic_3 (LifeStyle)	85.5	1.2	64.5^ac^	1.4	21.1^ac^	0.8
	FFP2_Aura	85.4	2.9	30.9^ab^	3.0	52.6^ab^	2.2
	FFP2_Palens	81.1	1.8	31.0^ab^	4.3	48.6^ab^	4.7
	FFP2_Biofield	81.6	2.3	30.1^ab^	3.0	50.4^ab^	2.5
	FFP3_MC002	83.7	1.7	13.4^bc^	0.8	68.0^bc^	1.7
High exercise	Surgical_1 (MVT)	125.1	5.9	20.6^bc^	8.4	95.5^bc^	7.2
	Surgical_2 (Radex)	123.8	3.3	28.7^bc^	1.6	89.7^bc^	3.2
	Hygienic_1 (Emotion)	116.6	3.7	92.4^ac^	3.2	22.9^ac^	0.9
	Hygienic_2 (Elite)	121.7	2.1	99.7^ac^	2.2	20.2^ac^	0.5
	Hygienic_3 (LifeStyle)	122.0	2.7	90.8^ac^	1.3	29.4^ac^	0.9
	FFP2_Aura	124.1	4.4	37.1^ab^	3.7	80.0*^b^	4.5
	FFP2_Palens	116.9	7.9	35.5^ab^	5.0	75.2*^b^	10.0
	FFP2_Biofield	117.3	2.0	35.7^ab^	3.2	77.7*^b^	2.0
	FFP3_MC002	120.7	1.6	15.0^bc^	0.3	98.4^bc^	1.3

*Significant differences with Surgical (MVT. Radex) p < 0.05.

**Significant differences with Hygienic (Emotion. Elite. LifeSytle) p < 0.05.

*** Significant differences with FFP2 (Aura. Palens. Biofield) p < 0.05.

^a^Significant differences with Surgical (MVT. Radex) p < 0.001.

^b^Significant differences with Hygienic (Emotion. Elite. LifeSytle) p < 0.001.

^c^Significant differences with FFP2 (Aura. Palens. Biofield) p < 0.001.

Figure 3 shows differences in filtered air between surgical facemasks and the rest of facemasks analyzed (p < 0.001), except for FFP3_MC002. The hygienic facemasks did not show differences in filtered air between them, but with all the others (p < 0.001). It can also be observed that the response of the FFP3 MC002 mask was more similar to that of the surgical than the FFP2 facemasks. FFP3 MC002 showed no statistical difference with the surgical facemask, while it showed differences with all the rest (p < 0.001). Moreover, the FFP2 facemasks filtered a very similar amount of air in all of them, with no differences between the three models analyzed.

**Figure 4:**
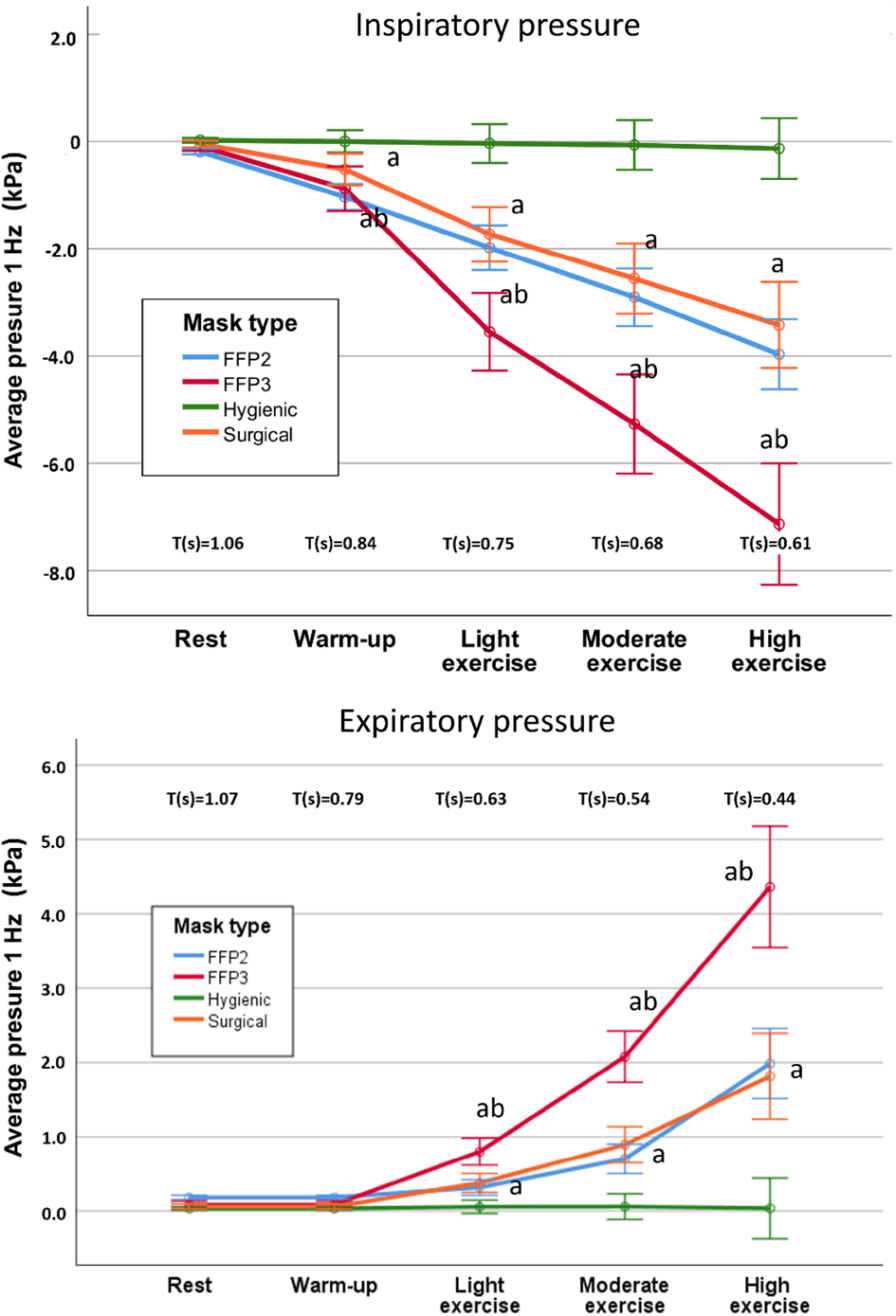
Inspiratory and expiratory pressures by exercise intensities, using median for discriminate. Below inspiratory and expiratory time (s) for each phase. The points indicate the mean value for each facemask group and the error bars 95% IC with statistical error of mean. (**a**) Significant differences with Hygienic (Emotion. Elite. LifeSytle) p < 0.001; (**b**) Significant differences with Surgical and FFP2 p < 0.001.

The analysis of the pressures is shown in Fig. 4. An interaction between the factors phase (intensity), breathing phase (inspiration/expiration) and type of facemask was found (F_(5,2,8)_ = 18, p < 0.001, ηp^2^ = 0.539). At all intensities, it can be observed that the pressure was higher in the inspiratory phase than in the expiratory phase. However, its response is not proportional but exponential.

It is also observed that the hygienic facemasks hardly increased the pressure necessary for the air to be filtered. On the contrary, there is an increase of pressure for the rest of the facemasks. Similar values were found for the surgical and the FFP2 facemasks. Finally, FFP3 mask presented a much greater resistance to airflow, both in inspiration and expiration processes (see Fig. 4).

Table 4 shows the most relevant results of the stepwise linear regression analysis, which allowed us to calculate the α-value. This table shows that almost all facemasks fail to reach 30% of filtered air that passes through them, while hygienic facemasks manage to filter 74.7% of that air.

**Table 4:** Results of linear regression analysis to obtain the coefficient of air filtered.

Equation of air filtered				R^2^	SEM	BFE	RBFE
	α-value						
AIR filtered (surgical) L/min	0.205	X	*AIRin*	0.774	4.469	97.0%	19.9%
AIR filtered (hygienic) L/min	0.786	X	*AIRin*	0.994	2.287	95.0%	74.7%
AIR filtered (FFP2) L/min	0.291	X	*AIRin*	0.891	3.845	95.0%	27.6%
AIR filtered (FFP3) L/min	0.112	X	*AIRin*	0.747	2.552	98.0%	11.0%

*BFE *average of bacterial filtration efficiency by facemask, *RBFE *real bacterial filtration efficiency, *AIRin *air input to the system for the Jaeger Oxycon Pro analyzer, *AIRfiltered *air filtered by the facemask for the Jaeger Oxycon Mobile analyzer.

## Discussion

As shown in the experiments reported, when the facemasks studied are worn, none of them allow the 100% of the air inspired or expired to pass through its filtering material. In addition, the air that passes through the facemask is highly dependent on the flow of inspiration/expiration and the facemask type. Therefore, the filtering capacity of the facemask cannot only be directly determined by the filtering capacity of the material it is made of, but the real filtering coefficient.

In accordance with our work, a previous study^17^ also showed large differences between facemasks: 98.5 ± 0.4% in the Fitted Filtration Efficiency (FFE) for the 3M1860N95 facemask and 71.5 ± 5.5% in the Fitted Filtration Efficiency (FFE) for the surgical facemask with strap adjustment and up to a value of 38.1 ± 11.4% in the Fitted Filtration Efficiency (FFE) for the surgical facemask with elastics on the ears.

However, previous attempts to analyze the air that passes through the facemask are typically grouped into two specific lines: qualitative methods, such as the ability to detect different chemicals by smell^18^; and quantitative methods, which analyze the particles that pass through the facemasks, due to the mismatch^17,19^. However, these systems only measure protection on inspiration, since they measure the entry of particles, but do not measure the exit. Nonetheless, most of the studies that analyze the filtration capabilities of the materials, including the fit of the facemask to the face, do it under conditions of rest^20^ or very light physical activity^17,18,21^. Therefore, they cannot be extrapolated to more intense situations. The natural airflows that occur naturally when breathing or talking range from 0.2 to 0.4 L/min. This would result in ventilations of 12 to 24 L/min, our data being within these natural ventilation ranges in humans^20^.

To solve this problem, this manuscript has created the Real Bacterial Filtering Efficiency (RBFE), assuming a correct perimeter adjustment. This adjustment could be a limitation of the study, as the weight of the protective plastic or even air redirection could influence the measurement^20^. The RBFE provides a correction factor for the facemasks, where the material is one of the most important aspects to consider for the correction, not only because of its filtration capability but its adjustment possibilities. If a wrong perimeter adjustment is done, the percentage of air that is filtered by any facemask decreases drastically. The RBFE has been elaborated based on 45 experiments carried out in a high-fidelity human face mannequin. However, there are some differences that should be considered. The mannequin is stiffer than a real human face and does not produce any facial movement, making the facemasks more difficult to condition but less prone to mismatch elasticity. Nonetheless, because of the protocol followed, the results allow to make a reliable comparison between different facemask’s types. On the other hand, the inclusion of experiments where particles or bacterial filtration can be analyzed could be definitive to clarify the real filtration of the masks and not that of the fabric from which they are made.

In the inspiratory phase, the absolute values of pressure are higher than in the expiratory phase. This has been described previously^22^ and would indicate that the pressure that the ventilation muscles must generate is not the same in the inspiratory phase than in the expiratory phase. This fact does not limit the work-performance but the worktime a person can execute^23^. This higher inspiratory pressure can be explained because during inspiration, the facemask adheres more to the surface of the face and the volume of filtered air is greater in the inspiratory phase than in the expiratory phase. This has an important practical consequence because inspired air is more filtered than expired air. This is demonstrated in Fig. 2, where the inspiratory and expiratory volumes are different, and this difference increases along with the intensity of the effort^24,25^.

In any case, the pressures generated depend, to a large extent, on the facemask used. The hygienic ones hardly produce an increase in pressure, possibly due to the characteristics of their fabric (highly breathable). The surgical ones have a significant effect on the increase in pressure, in the same way that the FFP2. Additionally, FFP3 facemasks increase resistance to airflow, without increasing the amount of filtered air. This agrees with previous studies^26^, which state that the pressure increases during inspiration, and that an increase in the resistance to the passage of air usually occurs in this phase.

However, it must not be forgotten that to correctly measure the characteristics of a facemask it is necessary to measure resistance to the passage of fluids, flammability, breathability, bacterial filtration, and particle filtration^18^, having shown large differences in breathability depending on the intensity of physical effort^24,25^. In fact, as the intensity increases, the respiratory reserve is affected^27^, and this could have an impact on the maximum exercise time that could be performed^24^.

The hygienic facemasks tested show statistical differences with respect to the rest of the facemasks tested. This finding is consistent with previous works related to its density and thickness^15^. This means that, at low airflows, rest and warm-up, the inspiratory and expiratory times are different, but as the intensity increases they converge. When the airflow is low and there is not enough air to be filtered by the facemask, the output sensor moves at a low speed and, therefore, the discretization times produce small artifacts. This effect disappears when the flow increases.

In general, the variables that are related to the flow show the filtration capacity of each type of facemask, and the time variables tend to be stable and without differences between analyzers. However, it should be noted that, at low airflow rates, the filtered and unfiltered air temporal variables are less accurate. This also occurs when the facemask offers a high resistance to air filtration and air volumes are low.

## Conclusions

The bacterial filtration efficiency of the facemasks analyzed in this study for the prevention of respiratory viruses is all overestimated since the filtration of the tissues is not that of the facemask when it is in used. On the other hand, this real filtration depends on whether the air is inspired or expired, and on the pressure necessary to breathe through them, having no relation to the real protection of the facemasks. Furthermore, the actual filtration depends on the type of respiration, being very different at rest or during exercise. Therefore, our results show that the real bacterial filtration efficiency of facemasks is well below what manufacturers claim in their specifications. As mentioned, throughout the document, very few studies have looked at a Real Bacterial Filtration Efficiency for different facemasks on the market. Although the most recent meta-analyses determine the usefulness of facemasks as protection against respiratory viruses^21^, the results presented in this manuscript should be a starting point to force the competent authorities to review, and modify if necessary, the current filtration standards for facemasks.

## Supplementary Information

Supplementary Information.

## Data Availability

The datasets used and/or analysed during the current study available from the corresponding author on reasonable request.
