# Supplementary material for: The real bacterial filtration efficiency to evaluate the effective protection of facemasks used for the prevention of respiratory diseases
Source: Sci Rep. 2023 Jun 5;13:8997. doi: 10.1038/s41598-023-35071-1 (PMC10241918; doi:10.1038/s41598-023-35071-1)
Supplement: Supplementary file 1 — Supplementary Information. [file 41598_2023_35071_MOESM1_ESM.docx]

**Supplementary data:**

**RELIABILITY FROM CONSECUTIVE PAIRS OF TRIALS**

Reference: Hopkins WG (2000). Reliability from consecutive pairs of trials using Excel spreadsheet.

The three gas analyzers (Vintus CPX, Oxycon Mobile and Oxycon PRO) were placed in serie, as shown in the image, pumping air with the calibration syringe at different intensities.


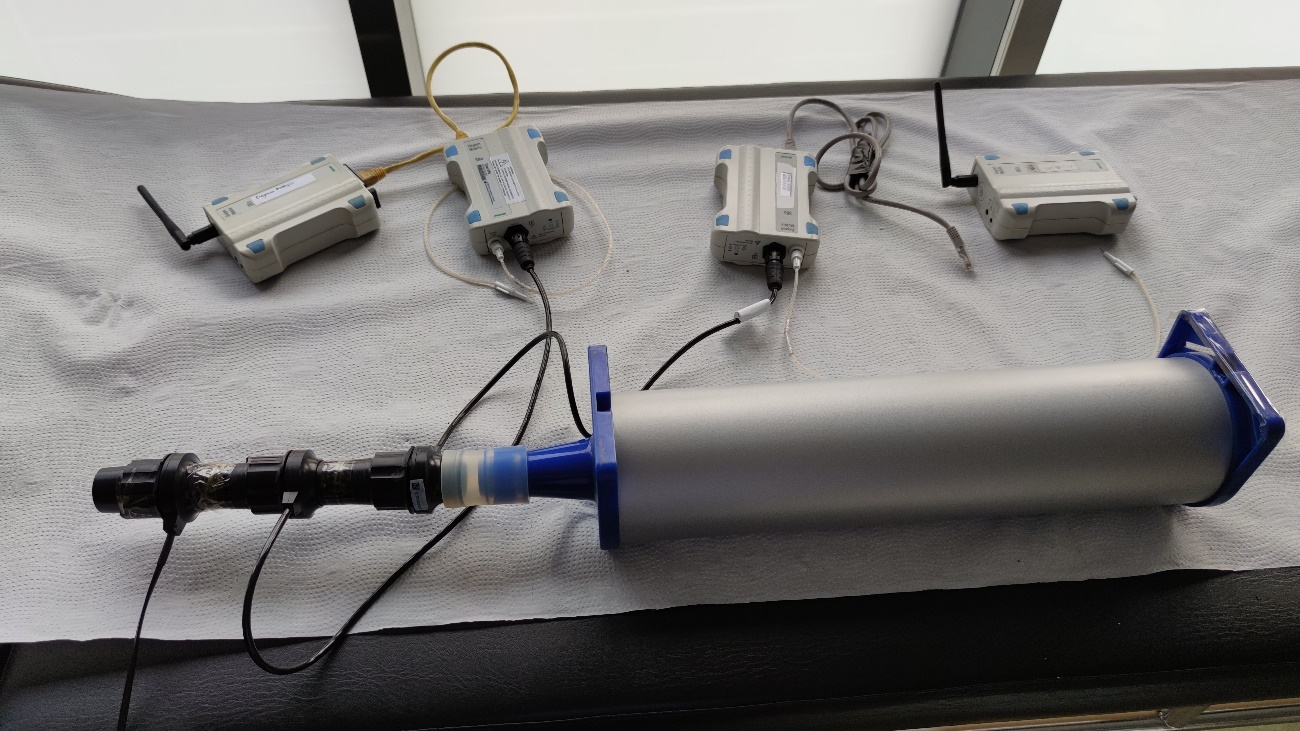


**
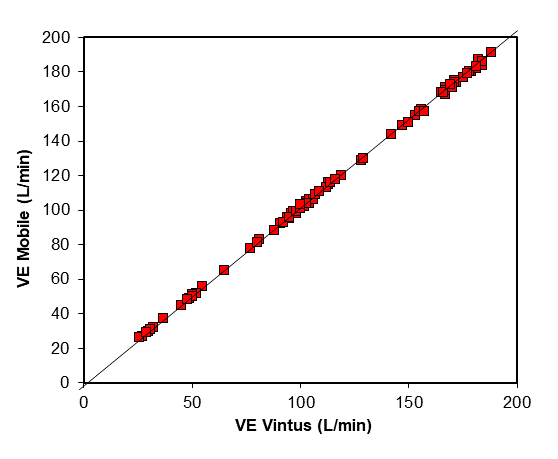

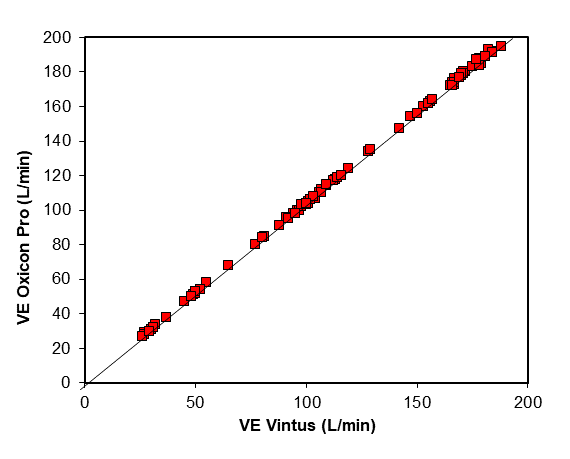
**

| **Measures of reliability for VE variable in liters** | | |
| --- | --- | --- |
|  | **Trial**  **Mobile-Vintus** | **Trial**  **Oxico Pro-Oxycon mobile** |
| **Change in mean** | **1.30** | **3.46** |
| Lower conf limit | 1.10 | 3.12 |
| Upper conf limit | 1.50 | 3.79 |
|  |  |  |
| **Typical error** | **0.82** | **1.35** |
| Lower conf limit | 0.73 | 1.21 |
| Upper conf limit | 0.93 | 1.55 |
| Degrees of freedom | 89 | 89 |
| TE^2 | 0.67 | 1.83 |
|  |  |  |
| Total error | 0.27 | 1.54 |
| Limits of agreement | 2.30 | 3.80 |
|  |  |  |
| Pearson r | 0.99985 | 0.99979 |
| Intraclass R | **0.99974** | **0.99932** |
| Lower conf limit | 0.99963 | 0.99904 |
| Upper conf limit | 0.99982 | 0.99952 |

**Total ICC R=0.999 with p<0.001.**

| **Effect of changing the plastic mask envelope for each measurement versus making all 5 measurements with the same plastic for the Hygienic_1, reusable (Emotion) mask. n=25.** | | | | | | | |
| --- | --- | --- | --- | --- | --- | --- | --- |
|  | | **Mean** | **SD** | **F** | **p-valor** | **t** | **p-valor** |
| **VE AIRin (L/min)** | No plastic change | 59.3 | 37.6 | 0.002 | 0.966 | -0.301 | 0.764 |
|  | Plastic change | 62.5 | 37.8 |  |  |  |  |
| **VE AIRfil (L/min)** | No plastic change | 46.9 | 29.9 | 0.04 | 0.842 | -0.099 | 0.922 |
|  | Plastic change | 47.7 | 29.0 |  |  |  |  |
| **VE AIRunf (L/min)** | No plastic change | 7.5 | 5.3 | 2.192 | 0.145 | -1.047 | 0.300 |
|  | Plastic change | 9.4 | 6.9 |  |  |  |  |
| AIRin= air input to the system for the Jaeger Oxycon Pro analyzer. AIRfil=air filtered by the facemask for the Jaeger Oxycon Mobile analyzer, and finally the Vintus CPX analyzer which collected the air that was not filtered (AIRunf). | | | | | | | |
